# Growth performance, organ-level ionic relations and organic osmoregulation of *Elaeagnus angustifolia* in response to salt stress

**DOI:** 10.1371/journal.pone.0191552

**Published:** 2018-01-23

**Authors:** Zhengxiang Liu, Jianfeng Zhu, Xiuyan Yang, Haiwen Wu, Qi Wei, Hairong Wei, Huaxin Zhang

**Affiliations:** 1 Research Center of Saline and Alkali Land of State Forestry Administration, Chinese Academy of Forestry, Beijing, P. R. China; 2 General Forestry Station of Beijing Municipality, Beijing, P. R. China; 3 School of Forest Resources and Environmental Science, Michigan Technological University, Houghton, Michigan, United States of America; Institute of Genetics and Developmental Biology Chinese Academy of Sciences, CHINA

## Abstract

*Elaeagnus angustifolia* is one of the most extensively afforested tree species in environment-harsh regions of northern China. Despite its exceptional tolerance to saline soil, the intrinsic adaptive physiology has not been revealed. In this study, we investigated the growth, organ-level ionic relations and organic osmoregulation of the seedlings hydroponically treated with 0, 100 and 200 mM NaCl for 30 days. We found that the growth characteristics and the whole-plant dry weight were not obviously stunted, but instead, were even slightly stimulated by the treatment of 100 mM NaCl. In contrast, these traits were significantly inhibited by 200 mM NaCl treatment. Interestingly, as compared with the control (0 mM NaCl), both 100 and 200 mM NaCl treatments had a promotional effect on root growth as evidenced by 26.3% and 2.4% increases in root dry weight, respectively. Roots had the highest Na^+^ and Cl^-^ concentrations and obviously served as the sink for the net increased Na^+^ and Cl^-^, while, stems might maintain the capacity of effective Na^+^ constraint, resulting in reduced Na^+^ transport to the leaves. K^+^, Ca^2+^ and Mg^2+^ concentrations in three plant organs of NaCl-treated seedlings presented a substantial decline, eventually leading to an enormously drop of K^+^/Na^+^ ratio. As the salt concentration increased, proline and soluble protein contents continuously exhibited a prominent and a relatively tardy accumulation, respectively, whereas soluble sugar firstly fell to a significant level and then regained to a level that is close to that of the control. Taken together, our results provided quantitative measures that revealed some robust adaptive physiological mechanisms underpinning *E*. *angustifolia*’s moderately high salt tolerance, and those mechanisms comprise scalable capacity for root Na^+^ and Cl^-^ storage, effectively constrained transportation of Na^+^ from stems to leaves, root compensatory growth, as well as an immediate and prominent leaf proline accumulation.

## Introduction

Soil salinity, as one of the major environmental adversities, has become increasingly problematic owing to its negative impacts on plant growth, productivity and even distribution [1], and also to its persistent spread and aggravation caused by global climate changes and various irrigation practices in many areas of the world [2–3]. It was conservatively estimated that the salt-affected land is approximately 1,000 million ha throughout the world, and in China, it is about 100 million ha (representing 10.4% of the total land area) and distributed primarily in the Northern China and secondarily in the east coastal regions [4–5]. If these salinized lands are not environmentally improved, they will further deteriorate, eventually impeding the economic development and ecological construction especially for Northern China [6]. Therefore, major research activities are carried out on unveiling the physiological and molecular mechanisms underlying salt tolerance [7–9], and selection and identification of salt-resistant plant species and cultivars [10–12], which have become the foci of plant breeders and biologists in recent years [13–15].

Plants in general show marked differences after being subjected to salt stress. The most discernible differences in phenotypes are often reflected in altered growth performance and dry weight accumulation that have been reported to vary from a prominent stimulation to a significant depression [7, 16]. The growth and dry weight of plant responses to salt stress can be intuitively exemplified using the following three patterns observed in three plant species: (1) *Arabidopsis thaliana* exhibited a sharp and continuous decline in relative shoot dry mass in response to increasing external salt concentrations [17], (2) *Distichlis spicata* presented an initial plateau (or a slight stimulation) and then a significant declination [18], whereas (3) *Suaeda maritime* performed a dramatic increase firstly and then a distinct reduction [19]. To date, the fundamental mechanisms of inhibitory plant growth at supra-optimal salinity levels remain elusive, though some plausible causes have been proposed [20–22].

Although some plant species can get rid of the absorbed ions by secretion, the majority of plant species have evolved a series of compartmentalization mechanisms at tissue, cell and organelle levels, to alleviate ionic toxicity. Qiu et al. [23] found that more than 70% of the total absorbed Na^+^ is distributed in *A*. *anethifolia* leaves, and that approximately 98% Na^+^ accumulated in leaves is localized in protoplasts. In *S*. *europaea*, the reserved Na^+^ is compartmentalized predominantly into vacuoles of endodermis cells in shoot [24]. Using two *S*. *salsa* populations originating from different saline environments as materials, Song et al. [25] compared the capabilities in root exclusion and leaf accumulation for Na^+^ and Cl^-^, and also investigated the distribution profiles of both ions at cellular level. In brief, ion homeostasis involving ionic uptake, transportation and compartmentalization plays crucial roles for plant growth and development under salt stress conditions.

*Elaeagnus angustifolia* L., a deciduous tree species in *Elaeagnus* genus, is distributed primarily in Northwest China and seldom in Northern China and west regions of Northeast China, where it is primarily located in the north of 34° N [5]. This species was also introduced to mid-east areas, including Shandong, Hebei and Tianjin for afforestation. *E*. *angustifolia* is widely and extensively planted in many marginal lands or environmentally harsh regions in Northern China owing to its exceptional endurance to salinization, wind, sand and barren. Although only reported as a typical halophyte [26], *E*. *angustifolia* has become one of the target species for studying salt tolerance [27], photosynthesis [28–29], water relations [30], anti-oxidation [31] and seed germination [32]. Despite its great value in afforestation on marginal lands, we are not aware of any quantitative investigation on growth and ion distribution to external salinity stress, needless to mention the intrinsically physiological and molecular mechanisms of salt tolerance.

In this study, solution-cultured *E*. *angustifolia* seedlings were employed to investigate the growth performances, ionic relations and compatible solute accumulation in response to salt stress under well-controlled greenhouse conditions. The following fundamental issues were expected to be expounded: firstly, which growth pattern the salt-stressed *E*. *angustifolia* seedlings belongs to among the aforementioned three? Secondly, do (does) Na^+^ and Cl^-^ accumulate in salt-stressed seedlings? If this is true, to what extent they accumulate, how they are partitioned at organ level, and how the salt ions affect other nutrient elements’ (K^+^, Ca^2+^ and Mg^2+^) contents and also their balance? Thirdly, which kinds of osmotic adjustment substances the salt-treated *E*. *angustifolia* seedlings critically evolve? It was hoped that the results of this study might provide novel insights for us to understand the adaptive physiology of this exceptional salt tolerance species.

## Materials and methods

### Plant materials

We obtained permissions from the Experimental Base (87°14’26” E, 44°6’4” N; Changji, Xinjiang Uyghur Autonomous Region, China) of Research Center of Saline and Alkali Land of State Forestry Administration, Chinese Academy of Forestry (CAF), to collect mature and healthy fruits of *E*. *angustifolia*. After the peels were detached and washed off, the seeds were air-dried and then stored in a refrigerator at 4°C until being used.

### Plant culture and salt treatments

After undergoing approximately three-month sandy stratification treatment in outdoors, *E*. *angustifolia* seeds were individually sown in plastic pots (8 cm diameter and 10 cm height) filled with vermiculite in the greenhouse of CAF, Beijing, China. After germination and growth for 14 days, the seedlings with the similar sizes, approximately 10 cm height and 8–10 leaves, were selected, and the vermiculite adhered to seedling roots were carefully and gently washed off. Then, the selected seedlings were transplanted to plastic pots of 40 cm length, 30 cm width and 15 cm height for hydroponics. The amount of 7 L nutrient solution used in this study was supplied every day with the following composition: 4 mM Ca(NO_3_)_2_·4H_2_O, 6 mM KNO_3_, 2 mM MgSO_4_·7H_2_O, 1 mM NH_4_H_2_PO_4_, 5 μM H_3_BO_3_, 0.25 μM ZnSO_4_·7H_2_O, 0.1 μM CuSO_4_·5H_2_O, 0.125 μM Na_2_MoO_4_·2H_2_O, 12.5 μM KCl, 0.5 μM MnSO_4_·H_2_O, 12.5 μM Fe-EDTA. The culture solution was replaced in every 4 days and aerated with a pump. The pH was adjusted to 6.0±0.1 with NaOH and/or H_2_SO_4_.

After pre-culturing for 18 days, 12 seedlings were harvested to determine the initial dry weight accumulation and ion contents (ultimately used to calculate the net accumulation of Na^+^ and Cl^-^), and the rest were subjected to different salt treatments of 0, 100 and 200 mM NaCl. Each treatment level included four replicate pots, and each pot contained 20 seedlings. The treatment solution was prepared by dissolving corresponding amount of NaCl in the above-mentioned nutrient solution. To avoid osmotic shock, salt was applied gradually by adding 50 mM NaCl per day. 200 mM NaCl treatment was applied two days earlier than 100 mM treatment so that the different treatments could reach the final concentrations on the same day, which was considered to be the initial date of salt treatment. The experiments were maintained for 30 days during which the greenhouse conditions included: natural sunlight with a 25±3°C air temperature regime, a relative humidity of 75±5%, and a carbon dioxide (CO_2_) concentration of 365±5 μmol mol^-1^. The dry weight accumulation and ion content were analyzed in oven-dried samples, whereas the growth parameters and osmolytes were determined in fresh samples and samples stored in liquid nitrogen, respectively.

### Measurement of growth parameters

At the end of the experiment, the dead plants were recorded to estimate plant survival rate, and eight survived seedlings per replication were randomly selected to determine growth indices and dry weight productivity. Stem heights and basal diameters, lateral branch numbers (recorded when its length ≥ 1 cm) and lengths were measured or counted.

For each seedling, the number of visibly damaged leaves (including abscission, chlorosis and necrosis) was counted, and then healthy leaves were immediately placed in sealed bags to determine growth parameters. Leaf area was measured for each seedling using a leaf area meter (Yaxin-1241, Beijing, China). A series of leaf growth parameters including leaf fresh weight (FW), number of functional leaves (fully expanded and area ≥ 1 cm^2^), leaf area per plant, area per leaf and specific leaf area were recorded, and leaf injury rate, area per leaf and specific leaf area were calculated respectively using the following formulae:

$$L\mathrm{eaf}\;\mathrm{injury}\;\mathrm{rate}=\frac{\mathrm{number}\;\mathrm{of}\;\mathrm{damaged}\;\mathrm{leaves}}{\mathrm{number}\;\mathrm{of}\;\mathrm{damaged}\;\mathrm{leaves}+\mathrm{number}\;\mathrm{of}\;\mathrm{functional}\;\mathrm{leaves}}\times 100\%$$

$$\mathrm{Area}\;\mathrm{per}\;\mathrm{leaf}=\frac{\mathrm{leaf}\;\mathrm{area}\;\mathrm{per}\;\mathrm{plant}}{\mathrm{number}\;\mathrm{of}\;\mathrm{functional}\;\mathrm{leaves}}$$

$$S\mathrm{pecific}\;\mathrm{leaf}\;\mathrm{area}=\frac{\mathrm{leaf}\;\mathrm{area}\;\mathrm{per}\;\mathrm{plant}}{\mathrm{leaf}\;\mathrm{dry}\;\mathrm{weight}\;\mathrm{per}\;\mathrm{plant}}$$

After the destructive harvest, seedlings were partitioned into leaves, stems and roots. All dry weight segments were dried separately in an oven at 105°C for 15 min and then at 80°C until reaching constant dry weight (DW). After that, root, stem, leaf and whole plant dry weight, root to shoot dry weight ratios (R/S), leaf water content (WC% = (1-DW/FW)×100), and organ dry weight allocation (= organ DW/plant DW×100%, organs include root, stem and leaf) were all recorded.

### Measurement of ion content

Approximately 0.1g DW sample was accurately weighted and placed into a glass beaker (50 mL) containing di-acid mixture of 10 mL HNO_3_ and 1 mL HClO_4_ and digested overnight. The solution was then placed on a hot-plate and evaporated to almost dryness at 100°C. The residues were dissolved in 0.5 mL of HNO_3_, and the inside walls of glass beakers were washed for 3–5 times using sub-boiling de-ionized water. After cooling down, a clear solution was obtained and finally diluted to 5 mL in volume. Na^+^, K^+^, Ca^2+^ and Mg^2+^ concentrations of different seedling organs were determined via Inductively Coupled Plasma Optical Emission Spectrometer (ICP-OES; Perkin Elmer Dual View Optima 5300DV, Waltham, MA, USA). The Cl^-^ concentrations were determined with a chloridometer (PXSJ-216, Leici Ltd, Shanghai, China) as described by Song et al. [25].

### Calculation of ionic relations

The net accumulation of *X* ion (Δ*X*_organ_, mg plant^-1^) in different plant organs was calculated using the following equation:

$$\Delta X_{\mathrm{organ}}={\left[X_{\mathrm{organ}}\right]}_{t_{1}}\times W_{\mathrm{organ},\;t_{1}}‑{\left[X_{\mathrm{organ}}\right]}_{t_{0}}\times W_{\mathrm{organ},\;t_{0}}$$

where, [*X*_organ]_ is the *X* ion (Na^+^ or Cl^-^) concentration in an organ (root, stem or leaf), mg g^-1^ DW; *W*_organ_ is the dry weight of corresponding organ, g plant^-1^; *t*_1_ and *t*_0_ are the initiation time (0 d) and termination time (30 d) of experiment. The net accumulation of *X* ion in shoots (Δ*X*_shoot_) and whole plants (Δ*X*_plant_), and the relative content of *X* ions in an organ (*X*_organ, RC_) (root, stem, leaf and shoot) were calculated using the following formulae:

$$\Delta X_{\mathrm{shoot}}=\Delta X_{\mathrm{stem}\;}+\;\Delta X_{\mathrm{leaf}}$$

$$\Delta X_{\mathrm{plant}}=\Delta X_{\mathrm{shoot}}+\;\Delta X_{\mathrm{root}}$$

$$X_{\mathrm{organ},\;\mathrm{RC}}=\frac{\Delta X_{\mathrm{organ}}}{\Delta X_{\mathrm{plant}}}\;\times \;100\%$$

The net uptake rate of *X* ion ($J_{\mathrm{plant}}^{X}$), and rate of *X* ion transport to the stem ($J_{\mathrm{stem}}^{X}$), to the leaf ($J_{\mathrm{leaf}}^{X}$) and to the shoot ($J_{\mathrm{shoot}}^{X}$) were then estimated using a previously reported equation [33]:

$$J_{\mathrm{organ}}^{X}=\frac{\Delta X_{\mathrm{organ}}\times \;\ln\;(W_{\mathrm{root},\;t_{1}}/W_{\mathrm{root},\;t_{0}})}{\left(t_{1}‑t_{0})\times (W_{\mathrm{root},\;t_{1}}‑W_{\mathrm{root},\;t_{0}}\right)}$$

where, $J_{\mathrm{organ}}^{X}$ is expressed as μmol g ^-1^ root DW d^-1^, and other parameters have the same meanings as mentioned above.

### Quantification of compatible solutes

Proline content and soluble sugar content were determined in fresh samples as described by Liu et al. [11]. For the extraction and determination of total soluble proteins, 200 mg of leaves were frozen in liquid N_2_ and ground in a grinder. After grinding, the fine powder was transferred to a 10-mL tube, 4 mL of extraction buffer (100 mM monobasic potassium phosphate, 1% polyvinylpyrrolidone-40 (PVP-40), 2 mM EDTA, pH 7.0) was added and then shaken vigorously for 30 s. Once the tissue was homogenized, the mixture was centrifuged at 10,000 g for 10 min at 4°C. The supernatant was removed and aliquoted in Eppendorf tubes and stored at -20°C until being used. Total soluble proteins were quantified by the dye-binding assay of Bradford [34] using bovine serum albumin (BSA) as a standard.

### Statistical analysis

Statistical analyses including analysis of variance (ANVOA) and multiple comparisons were performed using the program of PASW Statistics 18 (SPASS Inc., Chicago). All the acquired data were expressed as the mean (M) ± standard error (SE) of four (growth parameters) or three (ion content, ionic relations and osmolyte concentration) replications, and significant differences between the means were compared by Duncan’s multiple-range test. Unless otherwise stated, a difference was considered statistically significant when *P* < 0.05.

## Results

### Plant growth performance

*E*. *angustifolia* seedlings exhibited a negligible decline from 100% to 92.71% in survival rate as the salt concentration increased from 0 to 200 mM (Table 1). However, the leaf injury rate under 200 mM NaCl treatment was 15.47%, which was significantly higher than 3.71% and 6.22% injury rates corresponding to 0 and 100 mM salt treatments, respectively. Two stem traits, stem height and basal diameter, showed a similar changing trend, that is, both were markedly smaller under 200 mM than those under 0 or 100 mM NaCl. There was no significant difference of either of these two stem traits between 0 and 100 mM NaCl treatments (Table 1). It should be mentioned, however, that an approximate 6% increase of stem basal diameter was observed for the seedlings treated with 100 mM NaCl compared with the control. Salt treatment had an adverse effect on lateral branch growth as salt concentration increased. The number of lateral branches per plant had a statistically significant difference between any two of the three treatments. The length of lateral branches per plant under 200 mM NaCl treatment was dramatically shorter than those of the other two treatments, while the length of lateral branches per plant of 100 mM NaCl-treated seedlings was not significantly different when compared with that of the salt-free control.

**Table 1:** Effects of different salt treatments on growth performance of *Elaeagnus angustifolia* seedlings.

Treatments (mM)	Plant survival rate (%)	Leaf injury rate (%)	Stem height (cm)	Stem basal diameter (mm)	No. of lateral branches	Length of lateral branches (cm)
**0**	**100.00±0.00a**	**3.71±0.37b**	**59.40±1.01a**	**2.12±0.04a**	**5.25±0.54a**	**6.15±1.20a**
**100**	**96.88±3.12a**	**6.22±1.65b**	**56.03±1.18a**	**2.25±0.09a**	**2.75±0.49b**	**5.50±0.39a**
**200**	**92.71±3.56a**	**15.47±1.80a**	**39.45±1.38b**	**1.90±0.07b**	**0.90±0.06c**	**4.05±1.22b**

Values shown are the means (M) ± standard errors (SE) of four replicates (n = 4), different letters in each column indicate statistically significant differences among different salt treatments according to Duncan’s multiple-range test (α = 0.05).

### Leaf growth parameters

As shown in Table 2, the number of functional leaves and the total leaf area per plant were similarly and remarkably inhibited by salt stress. As salt concentrations increased from 100 to 200 mM, the functional leaf number dropped from 79.3% to 53.0% of the controls’, while the total leaf area per plant decreased from 92.4% to 52.2% of controls’. The differences in these two characteristics between the two salt concentrations were statistically significant.

**Table 2:** Effects of different salt treatments on leaf growth of *Elaeagnus angustifolia* seedlings.

Treatments (mM)	No. of functional leaves	Leaf area per plant (cm^2^)	Area per leaf (mm^2^)	Specific leaf area (cm^2^ g^-1^)	Leaf fresh weight (g)	Leaf water content (%)
**0**	**68.00±8.29a**	**170.65±10.98a**	**255.32±22.44b**	**402.48±13.22a**	**2.27±0.13a**	**81.26±2.22a**
**100**	**53.90±2.75a**	**157.65±14.57a**	**304.46±29.88a**	**376.25±15.72b**	**2.02±0.19a**	**79.06±1.26b**
**200**	**36.05±1.59b**	**89.12±4.92b**	**244.35±6.02b**	**317.04±14.12c**	**1.18±0.05b**	**76.03±1.37c**

Values shown are the means (M) ± standard errors (SE) of four replicates (n = 4), different letters in each column indicate statistically significant differences among different salt treatments according to Duncan’s multiple-range test (α = 0.05).

In terms of the area per functional leaf, a 19.2% increase and a 4.3% decrease were observed at 100 and 200 mM salt treatment, respectively, when compared with non-treated control. It is worth noting that area per functional leaf of 100 mM NaCl-treated seedlings was significantly larger than those of control and 200 mM NaCl-treated seedlings (Table 2). As the salt concentration increased from 0 to 100 and then to 200 mM, specific leaf area showed a consecutive and dramatic decline.

Leaf fresh weight and water content also decreased in a concentration-dependent manner (Table 2). The leaf fresh weight of 100 and 200 mM salt-treated seedlings showed a 10.8% and 47.9% reduction, respectively, as compared to the non-stressed controls, and there were significant differences in leaf water content between any two of the three salt treatment levels.

### Dry weight accumulation and allocation

There was a promotional effect of salt stress on root growth. Based on the data shown in Table 3, the root dry weight accumulation of 100 mM NaCl-treated seedlings was 26.3% higher than that of control, and even for the 200 mM NaCl concentration, there was also a 2.4% increase than that of control. Additionally, both 100 and 200 mM NaCl treatments inhibited stem, leaf and whole plant dry weight accumulation to different extents. Under the treatment of 100 mM NaCl, there was only a 6.2%, 1.0% and 0.8% decrease in stem, leaf and whole-plant dry weight in comparison with their corresponding controls, respectively, whereas the discrepancies in dry weight accumulation between different plant parts, including stems, leaves and whole-plant, of 200 mM NaCl-treated seedlings and their corresponding counterparts in control plants all reached significant levels, with a decline of 53.3% in stems, 33.5% in leaves, and 36.1% in whole plants.

**Table 3:** Effects of different salt treatments on dry weight accumulation and allocation of *Elaeagnus angustifolia* seedlings.

Parameters	Organs	Treatments (mM)		
		0	100	200
**Dry weight accumulation**	**Root (mg)**	**82.96±3.78b**	**104.75±6.97a**	**84.98±6.73b**
	**Stem (mg)**	**405.29±36.87a**	**380.14±29.16a**	**215.87±18.91b**
	**Leaf (mg)**	**426.28±28.10a**	**422.07±34.31a**	**283.69±16.83b**
	**Whole plant (mg)**	**914.52±63.98a**	**906.96±68.28a**	**584.54±40.61b**
**Dry weight allocation**	**R/S**	**0.1033±0.0033c**	**0.1317±0.0062b**	**0.1663±0.0070a**
	**Root (%)**	**9.13±0.32c**	**11.60±0.52b**	**14.53±0.55a**
	**Stem (%)**	**44.22±0.85a**	**41.90±0.50a**	**36.80±0.86b**
	**Leaf (%)**	**46.65±0.64a**	**46.50±0.63a**	**48.68±1.28a**

Values shown are the means (M) ± standard errors (SE) of four replicates (n = 4), different letters in each row indicate statistically significant differences among different salt treatments according to Duncan’s multiple-range test (α = 0.05).

Salt stress also changed the dry weight distribution pattern. Both root/shoot dry weight ratio (R/S) and root dry weight percentage showed an upward trend with the increase of salt concentration (Table 3). As the NaCl concentration increased from 0 to 200 mM, R/S and the root dry weight allocation increased from 0.1033 to 0.1663, and from 9.13% to 14.53%, respectively, whereas the stem dry weight distribution decreased from 44.22% to 36.80%. The leaf dry weight allocation was relatively steady and maintained between 46.5% and 48.7%, no matter the concentration of NaCl was 0, 100 or 200 mM.

### Ion content in different organs

**Fig 1:**
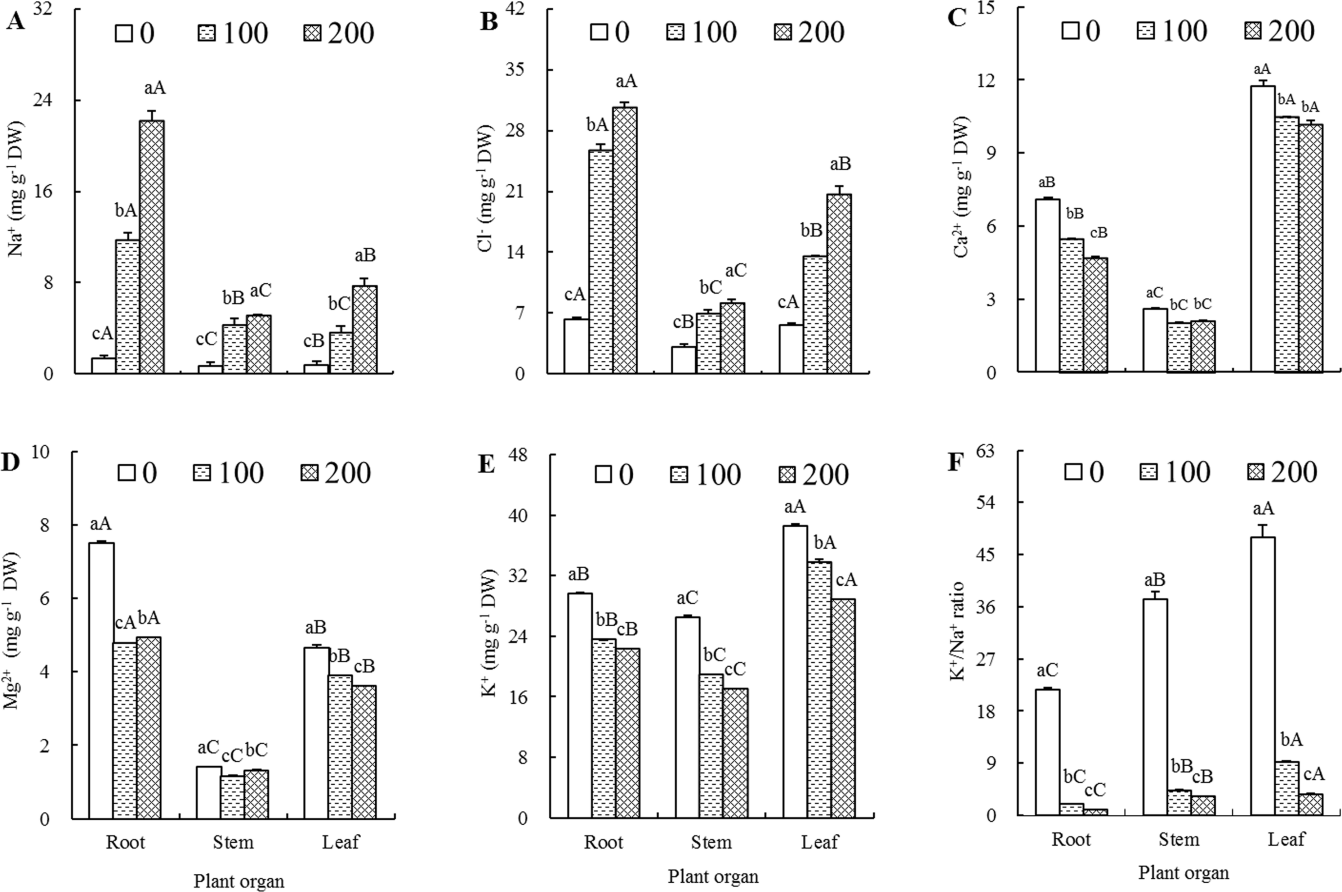
Effects of different salt treatments on ion contents of *Elaeagnus angustifolia* seedlings. Na^+^ (A), Cl^-^ (B), Ca^2+^ (C), Mg^2+^ (D), K^+^ (E) concentrations and K^+^/Na^+^ ratios (F) were determined in roots, stems and leaves of *E*. *angustifolia* seedlings hydroponically treated with 0, 100 and 200 mM NaCl for 30 days. Each vertical bar represents standard error (SE) for the mean (M) of three replications (n = 3). Different letters above the vertical bars denote the significant levels at 0.05 according to Duncan’s multiple-range test (A-C denote significance between the different plant organs at a given salt concentration, whereas a-c denote significance between the different salt concentrations in a given plant organ).

The Na^+^ concentrations in various plant organs exhibited a drastically increasing tendency during the progress of salt treatment. The differences between any two of the three salt treatment levels (0,100 and 200 mM NaCl) in a given plant organ (roots, stems or leaves) and the differences between any two of the three plant organs (roots, stems and leaves) at a given salt treatment level (0,100 or 200 mM NaCl) were all above the significant level (Fig 1A). As the NaCl concentration increased from 0 to 100 and then to 200 mM, the Na^+^ concentrations in roots, stems and leaves of salt-treated seedlings were 8.56, 6.03 and 4.50 times at 100 mM NaCl, and 16.20, 7.22 and 9.58 times at 200 mM NaCl compared to those of their respective controls. These data clearly demonstrated that NaCl-treated seedlings absorbed and accumulated a great deal of Na^+^, and that the majority of Na^+^ was sequestered in root organ. As also shown in Fig 1A, the Na^+^ concentrations from high to low in 0, 100 and 200 mM NaCl-treated seedlings were roots > leaves > stems, roots > stems > leaves and roots > leaves > stems, respectively, indicating that salt stress changed the order of Na^+^ contents among different plant organs.

Changes of Cl^-^ during salt treatment had a similar story with Na^+^ (Fig 1B). The Cl^-^ concentrations in roots, stems and leaves of 100 mM NaCl-treated seedlings were 4.06, 2.27 and 2.43 times higher than those of control seedlings, respectively. The Cl^-^ concentrations in roots, stems and leaves of 200 mM NaCl-treated seedlings were 4.84, 2.68 and 3.70 times higher than those of their respective control seedlings. The organ Cl^-^ concentrations from high to low in 0, 100 and 200 mM NaCl-treated seedlings showed the same order, that is, roots > leaves > stems. In general, the Cl^-^ concentrations in various plant organs were much higher than the Na^+^ concentrations in the corresponding organs at the same salt stress level (Fig 1A and 1B). Nevertheless, it was noted that the magnitude of Na^+^ increase was much larger than that of Cl^-^ when compared with their corresponding controls.

In general, Ca^2+^, Mg^2+^ and K^+^ concentrations in various plant organs exhibited a similar pattern, that is, all showed a downward trend with the increase of salt treatment concentration (Fig 1C–1E). When the salt treatment concentrations increased from 0 to 100 and then to 200 mM, root Ca^2+^ content, leaf Mg^2+^ content, and K^+^ contents in roots, stems and leaves presented consecutive and prominent decreases. Mg^2+^ contents in both root and stem organs showed a drop first and then an increase (Fig 1D). However, there was no statistically significant difference in both stem and leaf Ca^2+^ contents between 100 and 200 mM salt treatment levels (Fig 1C). As for ion contents among different organs, it was easily observed that there were all significant differences in Ca^2+^, Mg^2+^ and K^+^ contents at a given salt treatment level (0, 100 or 200 mM NaCl) between any two of the three plant organs (roots, stems and leaves) (Fig 1C–1E). The Ca^2+^ and K^+^ contents from high to low in all three treatment levels were leaves > roots > stems, whereas Mg^2+^ content was roots > leaves > stems.

An increase in Na^+^ content and a decrease in K^+^ content resulted in a decrease in K^+^/Na^+^ ratio (Fig 1F). Statistical analysis indicated that there were all significant differences in K^+^/Na^+^ ratios between any two of the three salt treatment levels in a given plant organ and between any two of the three plant organs at a given salt treatment level. For any one of the three salt treatment levels, the K^+^/Na^+^ ratio was the highest in leaves, medium-leveled in stems, and lowest in roots.

### Net accumulation and relative distribution of Na^+^

**Fig 2:**
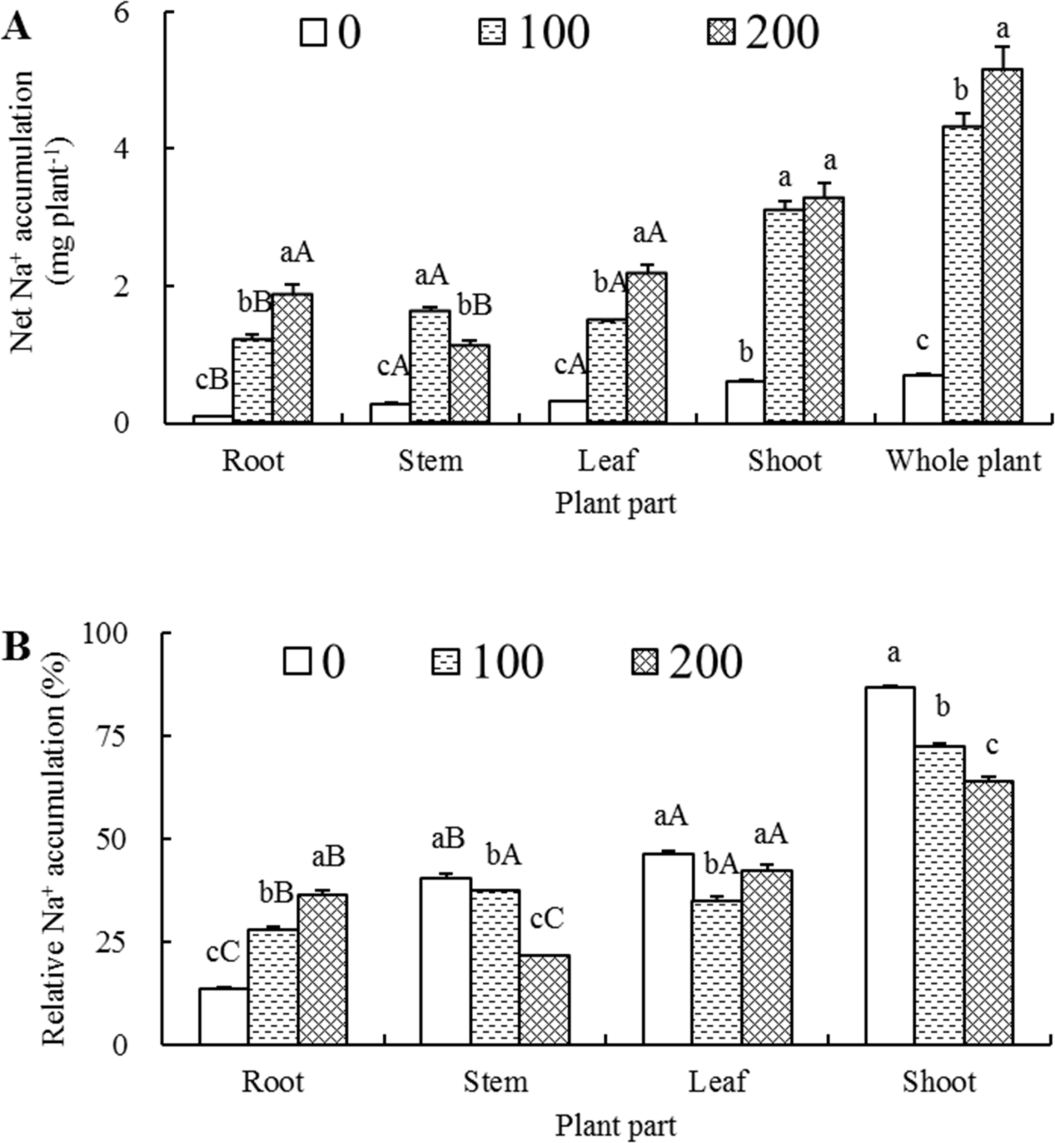
Effects of different salt treatments on Na^+^ accumulation and distribution of *Elaeagnus angustifolia* seedlings. Net Na^+^ accumulation (A) and relative Na^+^ accumulation (B) were determined in roots, stems, leaves, shoots and/or whole plant of *E*. *angustifolia* seedlings hydroponically treated with 0, 100 and 200 mM NaCl for 30 days. Each vertical bar represents standard error (SE) for the mean (M) of three replications (n = 3). Different letters above the vertical bars denote significant levels at 0.05 according to Duncan’s multiple-range test (A-C denote significance between the different plant organs at a given salt concentration, whereas a-c denote significance between the different salt concentrations in a given plant organ).

With the increase of salt treatment concentration, the net Na^+^ in roots, leaves, shoots and whole plant accumulated successively, whereas in stems it exhibited a drastic increase first and then a significant decline, but still markedly higher than that of control (Fig 2A). When compared to their respective non-saline controls, the net Na^+^ accumulations in 100 and 200 mM NaCl-treated seedlings increased 12.87 and 20.06 times in roots, 5.73 and 3.94 times in stems, 4.66 and 6.77 times in leaves, 5.16 and 5.45 times in shoots, and 6.19 and 7.41 times in whole plants compared to those in the corresponding organs of control seedlings, respectively. The organ net Na^+^ accumulations from high to low in 0, 100 and 200 mM NaCl-treated seedlings were leaves > stems > roots, stems > leaves > roots and leaves > roots > stems, respectively, whereas the increasing magnitudes of net Na^+^ accumulations in roots, leaves and stems were averagely 16.47, 5.72 and 4.84 times in comparison with their respective controls. As also shown in Fig 2A, when the NaCl concentration increased from 100 to 200 mM, the net Na^+^ accumulation increased 19.6% in whole plants, whereas it increased only 5.6% in shoots, indicating that the root was the primary organ for net Na^+^ accumulation.

To elucidate the distribution of net Na^+^ accumulation from a different angle, we calculated the relative percentage of Na^+^ content in each plant part with respect to the whole plant. As shown in Fig 2B, when the NaCl concentration increased from 0 to 100 and finally to 200 mM, root relative Na^+^ content increased significantly from 13.4% to 27.8% and then to 36.2%. In contrast, stem relative Na^+^ content decreased significantly from 40.5% to 37.5% and then to 21.5%, and shoot relative Na^+^ content decreased from 86.6% to 72.3% and then to 63.8%. In leaves, the relative Na^+^ content first declined from 46.2% to 34.8%, and then restored to 42.3%. The relative Na^+^ contents of 0, 100 and 200 mM NaCl-treated seedlings were leaves > stems > roots, stems > leaves > roots, and leaves > roots > stems, respectively, indicating that roots play an increasingly vital role in Na^+^ relation during the progressively increased salinization.

### Net accumulation and relative distribution of Cl^-^

**Fig 3:**
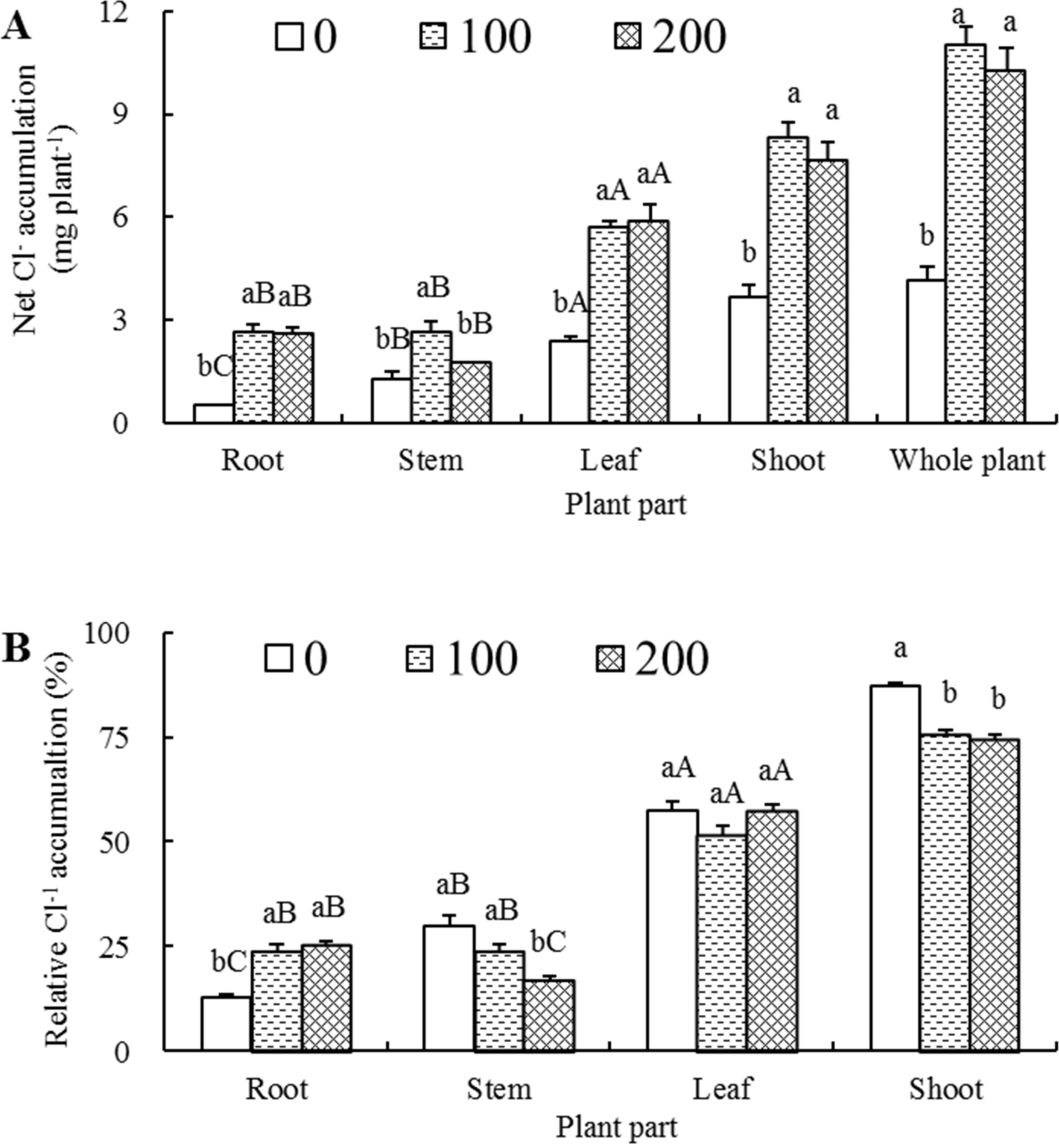
Effects of different salt treatments on Cl^-^ accumulation and distribution of *Elaeagnus angustifolia* seedlings. Net Cl^-^ accumulation (A) and relative Cl^-^ accumulation (B) were determined in root, stem, leaf, shoot and/or whole plant of *E*. *angustifolia* seedlings hydroponically treated with 0, 100 and 200 mM NaCl for 30 days. Each vertical bar represents standard (SE) for the mean (M) of three replications (n = 3). Different letters above the vertical bars stand for significant levels at 0.05 according to Duncan’s multiple-range test (A-C denote significance between the different plant organs at a given salt concentration, whereas a-c denote significance between the different salt concentrations in a given plant organ).

Under 100 and 200 mM NaCl stress conditions, the net Cl^-^ accumulations in roots, stems, leaves, shoots and whole plants were all significantly higher than those of their respective controls, with an exception of the stems of the seedlings treated with 200 mM NaCl, where the net Cl^-^ accumulation was not statistically significant but increased 40.4% (Fig 3A). The net Cl^-^ accumulations in the roots, stems and leaves of 100 and 200 mM NaCl-treated seedlings were averagely 5.02, 1.75 and 2.43 times higher than those of their respective controls. When the salt concentration increased from 100 to 200 mM, the net Cl^-^ accumulations in roots and leaves remained almost unchanged, whereas in stems, it decreased significantly (33.2%), leading to 8.0% and 6.7% declines in shoots (stems + leaves) and whole plants, respectively (Fig 3A).

Compared with non-saline controls, salt treatment induced a significant increase of the relative Cl^-^ content in roots, a drastic decline in both stems and shoots, and a slight fluctuation in leaves (Fig 3B). The relative Cl^-^ contents of 0, 100 and 200 mM NaCl-treated seedlings were leaves > stems > roots, leaves > roots > stems, and leaves > roots > stems, respectively, indicating that leaf is the primary distribution organ of net Cl^-^ accumulation.

### Net uptake and transport rates of Na^+^ and Cl^-^

**Fig 4:**
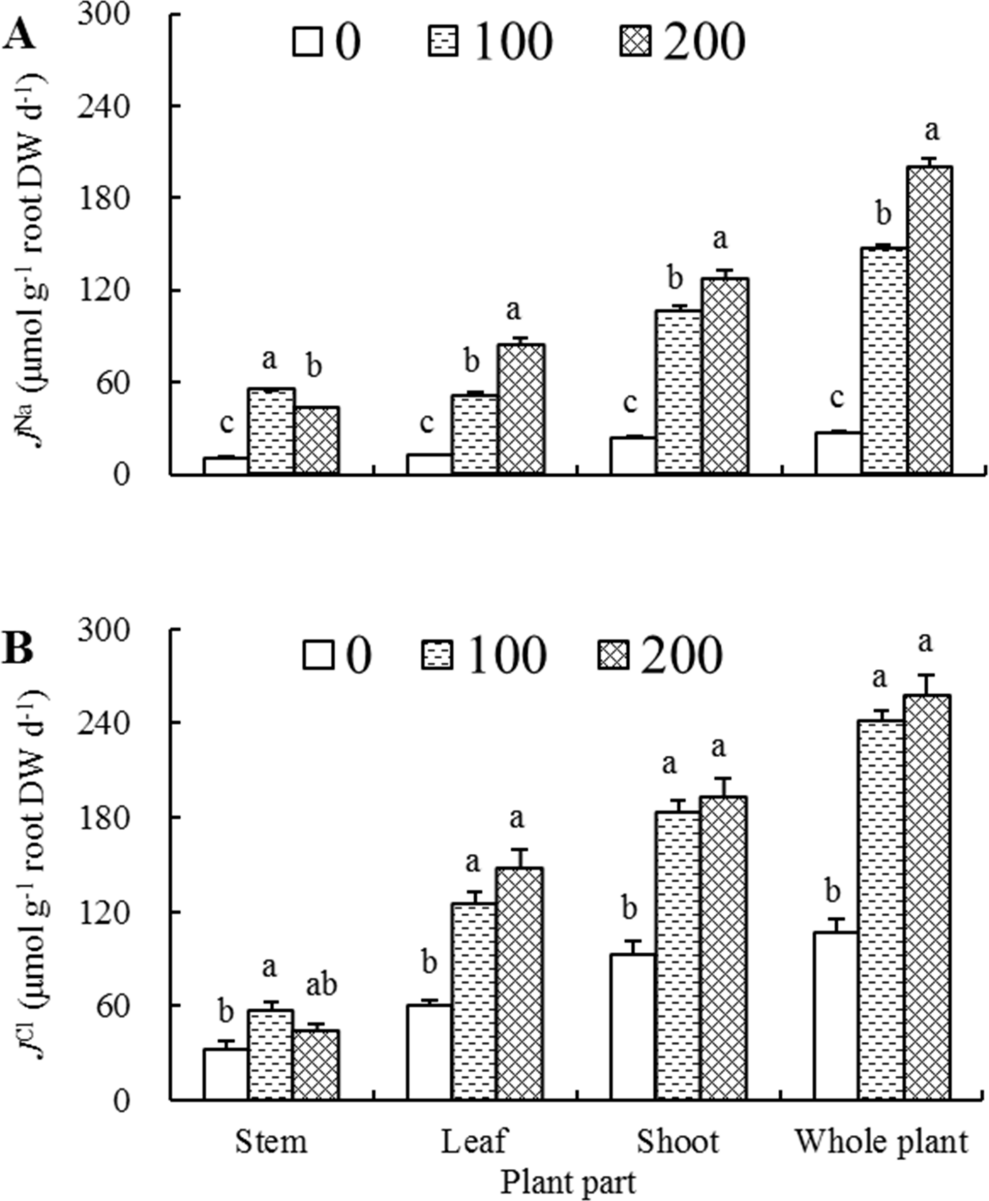
Effects of different salt treatments on net ion rates of *Elaeagnus angustifolia* seedlings. Net Na^+^ rates (*J*^Na^, A) and net Cl^-^ rates (*J*^Cl^, B) were determined in stem, leaf, shoot and whole plant of *E*. *angustifolia* seedlings hydroponically treated with 0, 100 and 200 mM NaCl for 30 days. Each vertical bar represents standard error (SE) for the mean (M) of three replications (n = 3). Different letters above bars denote significant levels between the different salt concentrations in a given plant part at 0.05 according to Duncan’s multiple-range test.

It was conspicuous that net ion uptake or transport rates exhibited an increasing tendency as salt concentration increased irrespective of tested ions and plant organs, with the exception of stem in which both *J*^Na^ and *J*^Cl^ had a dramatic increase first and then a significant or slight decline, but both were still higher than those of corresponding controls (Fig 4). The net Na^+^ rates in all plant parts were generally lower than net Cl^-^ rates in the comparable plant parts. However, the increasing magnitude of net rates responding to salt stress differed between the two kinds of ions. Compared with the corresponding controls, $J_{\mathrm{stem}}^{\mathrm{Na}}$, $J_{\mathrm{leaf}}^{\mathrm{Na}}$, $J_{\mathrm{shoot}}^{\mathrm{Na}}$ and $J_{\mathrm{plant}}^{\mathrm{Na}}$ of 200 mM NaCl-treated seedlings increased 2.87, 5.67, 4.36 and 6.28 times, respectively, whereas the net Cl^-^ rates increased only 0.39, 1.45, 1.08 and 1.43 times, respectively.

Under 0 and 100 mM NaCl treatment conditions, $J_{\mathrm{stem}}^{\mathrm{Na}}$ was comparable to $J_{\mathrm{leaf}}^{\mathrm{Na}}$ (Fig 4A). The *J*^Na^ of 100 mM NaCl-treated seedlings decreased from 54.84 μmol g^-1^ root DW d^-1^ in stems to 50.94 μmol g^-1^ root DW d^-1^ in leaves, whereas in 200 mM NaCl-treated seedlings, the *J*^Na^ increased from 42.92 μmol g^-1^ root DW d^-1^ in stems to 84.31 μmol g^-1^ root DW d^-1^ in leaves, indicating that at lower saline condition of 100 mM NaCl, Na^+^ was preferentially transported to stems in comparison with leaves, while at a higher external NaCl concentration of 200 mM, much more Na^+^ were transported to leaves than those transported to stem organ. As to Cl^-^, under 100 and 200 mM NaCl treatment conditions, $J_{\mathrm{leaf}}^{\mathrm{Cl}}$ were both greatly higher than $J_{\mathrm{stem}}^{\mathrm{Cl}}$, averaging 137.07 and 51.19 μmol g^-1^ root DW d^-1^, respectively (Fig 4B). However, in both organs there were no statistical difference in *J*^Cl^ between 100 and 200 mM NaCl-treated seedlings, indicating that much more amount of Cl^-^ was transported to leaves than those transported to stems under two saline conditions, and that *J*^Cl^ maintained a relatively stable level in both organs when salt concentration increased from 100 to 200 mM.

### Compatible solutes content

**Fig 5:**
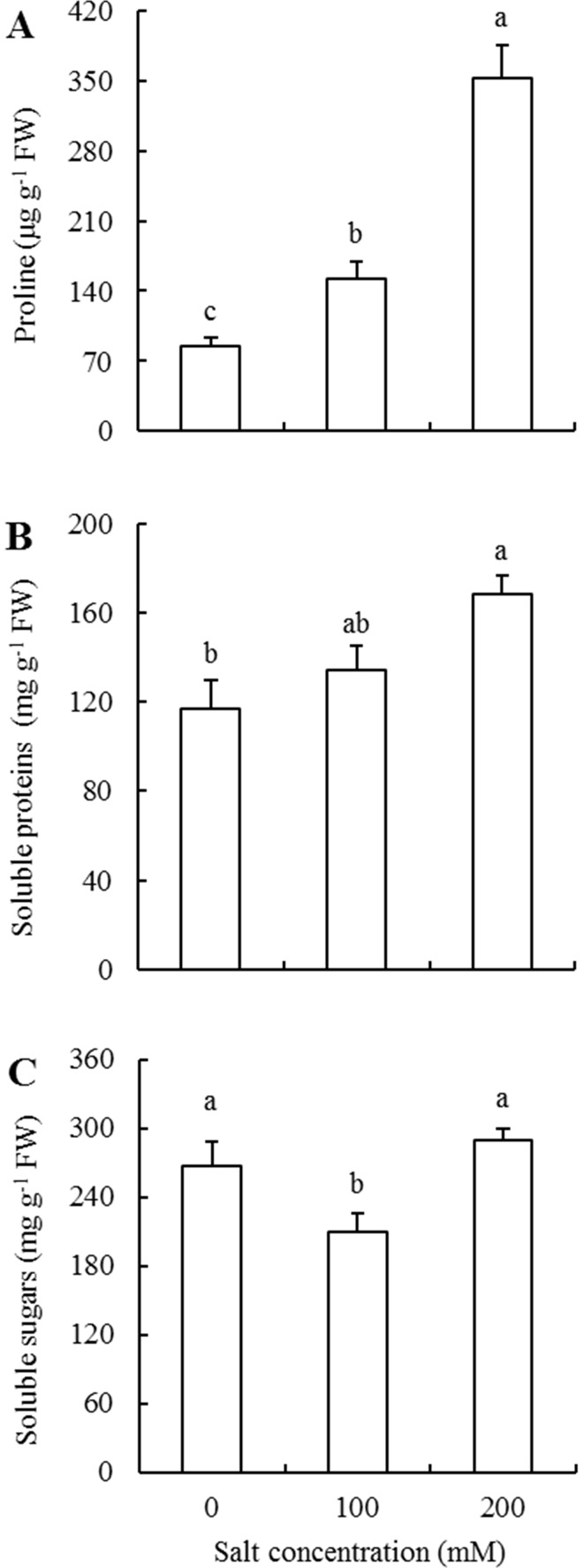
Effects of different salt treatments on osmolyte accumulation of *Elaeagnus angustifolia* seedlings. Proline (A), soluble protein (B) and soluble sugar (C) contents were determined in leaves of *E*. *angustifolia* seedlings hydroponically treated with 0, 100 and 200 mM NaCl for 30 days. Each vertical bar represents standard error (SE) for the mean (M) of three replications (n = 3). Different letters above bars denote significant levels between the different salt concentrations at 0.05 according to Duncan’s multiple-range test.

Proline content in leaves of 100 and 200 mM NaCl-treated plants showed a sequential and significant increase over those of their respective control plants (Fig 5A). The proline contents of 100 and 200 mM NaCl-treated seedlings increased a 1.79-fold and 4.14-fold as compared to that in control seedlings. In addition, the soluble proteins in 200 mM salt treatment seedlings increased 1.45-fold, and there were no significant differences between 100 mM and the other two salt treatment levels (Fig 5B). Whereas, as the salt concentration increased from 0 to 100 and then to 200 mM, soluble sugar content changed from 265.79 to 210.10 and then to 289.95 mg g^-1^ FW, exhibiting a notable decline first and then a significant increase to the level higher than that of control (Fig 5C).

## Discussion

### Growth performance of *E*. *angustifolia* under saline conditions

Inhibitory growth is a typical physiological response of most plant species to saline environmental conditions [35]. Nevertheless, all halophytes and some glycophytes often manifest stimulative rather than inhibitive growth and dry weight accumulation under certain levels of salt treatment [7,16]. As demonstrated, 100 mM NaCl treatment in general had no evidently inhibitory effects on *E*. *angustifolia*’s growth, and various degrees of promotions were even observed for some traits (Tables 1–3). This is consistent with the results obtained from other salt-tolerant plant species those include *Phragmites karka* and *Spartina maritima* [36–37]. In contrast, 200 mM NaCl treatment had a significantly adverse effect on whole plant growth performance, but even so the plant survival rate was maintained as high as 92.7%, providing quantitative measures those are indicative of *E*. *angustifolia*’s moderately high salt tolerance. The similarities made us speculate that *E*. *angustifolia* owns a comparable salt tolerance with *Thellungiella halophile*, which held a 55% whole-plant relative dry weight at 200 mM NaCl-treated conditions [38] and presented a relatively coincident growth performance between 100 mM NaCl-treated and control plants [39].

Stressed plants could accommodate and survive the adversities by changing its dry weight distribution model [16]. Accordingly, root/shoot dry weight ratio (R/S) and their allocation percentage are the two key indices that have been used to evaluate to what extent plants are influenced by adverse environments [40–41]. In this study, 100 and 200 mM NaCl treatment, especially 100 mM, had a stimulant effect on root growth and development as shown by increased dry weight accumulation and allocation (Table 3). Abideen et al. [36] and Wang et al. [42] reported that extensive root growth could compensate their absorption function loss resulting from salt treatment by dry weight accumulation and consequently superficial area enlargement, and those compensation effects are of great importance for stressed plants to maintain normal growth and water demand of aboveground shoots and to help in the absorption of K^+^ and storage of Na^+^ in roots. We speculated that root compensatory growth might be one mechanism that augments salt tolerance in *E*. *angustifolia*.

### Organ-level ionic relations revealed some physiological mechanisms of *E*. *angustifolia* under saline conditions

In this study, the Na^+^ and Cl^-^ concentrations in roots were tremendously higher than those in stems and leaves at both 100 and 200 mM NaCl levels (Fig 1A and 1B), the magnitudes of both net Na^+^ and Cl^-^ accumulation in roots were the highest among three organs though root was not the highest organ that had the highest net accumulation owing to its relatively small dry weight accumulation (Figs 2 and 3), indicating that root Na^+^ and Cl^-^ storage play vital roles in salt tolerance of *E*. *angustifolia*. These results are in overall aligned well with the results from *P*. *karka* [36], *Quercus virginiana* [42] and *Aeluropus lagopoides* [43].

Yet, the most interesting information provided by these experiments is probably that, under 100 mM NaCl treatment, the concentration, accumulation and distribution, and transport rate of Na^+^ in stems were, to a different extent, higher than those in leaves (Figs 1A, 2 and 4A); whereas, for 200 mM NaCl-treated seedlings, these parameters in leaves were all significantly higher than those in stems. These results indicated that stems could store and sequestrate Na^+^ for mitigating Na^+^ toxicity in leaves under lower level salt treatment; whereas, under the higher level salt treatment, the stem appeared to accumulate Na^+^ to a level beyond its maximum tolerable magnitude so that more amount of Na^+^ would be transported and allocated to leaves. These results, on the one hand, were corresponded to unnoticeably stunted growth characteristics of 100 mM NaCl-treated seedlings, and a significant decrease in dry weight accumulation and a prominently worse growth performance under 200 mM NaCl (Tables 1–3). On the other hand, these conclusions were also accordant with the results obtained by Jaarsma et al. [44], who found that salt-tolerant potato (*Solanum tuberosum*) cultivars maintained the capacity of stem Na^+^ accumulation, leading to a reduced Na^+^ transportation to leaf organ. So it might be presumed that effectively constrained transportation of Na^+^ from stems to leaves play a vital role in *E*. *angustifolia* salt tolerance.

In general, NaCl-treated plants accumulate more or less amount of Cl^-^ when synchronously absorb a certain amount of Na^+^ in order to partially maintain an internal charge homeostasis [8], and Cl^-^ transport is also correlated closely with plant salt tolerance [45]. In this study, we found that as the NaCl concentrations increased from 100 to 200 mM, net Cl^-^ transport rates, unlike net Na^+^ transport rates which elevated significantly in shoots and whole plants, increased only 5.1% and 6.6% in shoots and whole plants, respectively (Fig 4), indicating that *E*. *angustifolia* might deploy some mechanisms to exclude Cl^-^ entry and/or discharge entered Cl^-^. This is similar to what were found in *Vitis* species [46], *Malus hupehensis* [47] and *Poncirus trifoliate* [48].

### Organic osmoregulation is important for salt tolerance in *E*. *angustifolia*

*E*. *angustifolia* had considerably lower water content in leaves when exposed to increasing NaCl concentration (Table 2). This is possibly due to a reduction in transpiration rate [28], which resulted in turgor loss [36]. To maintain and re-establish cytoplasmic homeostasis after being subjected to the salt-induced osmotic stress, some plants immediately administer osmotic adjustment involving the production and accumulation of organic compatible solutes [49]. A sharp and a relatively tardy accumulation of proline and soluble proteins was, respectively, exhibited upon increasing salt stress intensity (Fig 5A and 5B), indicating that proline and soluble proteins are the two important osmolytes in salt-treated *E*. *angustifolia* seedlings and that proline appears to play much more roles in osmotic adjustment.

As for soluble sugar content, it fell significantly at the initial stage and then reverted to a slightly higher level than that of the control (Fig 5C). Similar to the results in this study, a decreasing soluble sugar concentration was also reported in *Rhus glabra* [11] and *Vicia faba* [50] in response to salt treatment. It was deduced that salt-stressed *E*. *angustifolia* might provide sufficient carbon skeleton and energy for seedling growth by increasing sugar degradation at 100 mM NaCl stress conditions [51], and this assertion, on the other hand, was supported by a slightly stimulated growth performance in this study (Tables 1–3).

### Conclusions

This is the first report of quantitative growth analysis, relatively comprehensive organ-level ionic relation investigation and organic osmolyte determination of *E*. *angustifolia* upon being subject to different salinity stress regimes. The acquired results provided quantitative measures that manifested some physiological mechanisms underlying *E*. *angustifolia*, a species with moderately high salt tolerance and some halophytes-like characteristics. One strategy *E*. *angustifolia* employed to combat salt stress is to increase root capacity by stimulating root growth under 100 mM NaCl stress while adopting growth scale reduction to counteract 200 mM NaCl stress. As a result, root Na^+^ and Cl^-^ storage and stem Na^+^ effectively constrained transportation to functional leaves were revealed as the primary physiological mechanisms underlying the salt tolerance in *E*. *angustifolia*. This is different from the mechanisms in *A*. *anethifolia* and *S*. *europaea*, which stores majority of Na^+^ in leaves and vacuoles of endodermis cells in shoot, respectively. Accompanied with the decline in photosynthesis, an immediate and sharp proline accumulation may also play vital roles in re-establishment of cytoplasmic homeostasis upon salt-induced osmotic stress. Therefore, future research should focus on unlocking the underlying physiological mechanisms in root tolerance and stem sequestration under high Na^+^. As one of the moderately salt-tolerant tree species with some unique characteristic, *E*. *angustifolia* can be studied as a model to learn salt-tolerance pathways and asymmetric ion transport in woody species, which can serve as a sink with much large capacity for cultivation of the extensive saline lands.

## References

[1] Al Hassan M, Pacurar A, López-Gresa MP, Donat-Torres MP, Llinares JV, Boscaiu M, et al Effects of salt stress on three ecologically distinct *Plantago* species. PLoS ONE. 2016; 11(8): e0160236 doi: 10.1371/journal.pone.0160236 27490924 10.1371/journal.pone.0160236 PMC4973956

[2] Li L, Zhang YB, Luo JX, Korpelainen H, Li CY. Sex-specific responses of *Populus yunnanensis* exposed to elevated CO_2_ and salinity. Physiol Plantarum. 2013; 147(4): 477–488. 10.1111/j.1399-3054.2012.01676.x 22897484

[3] Chen SL, Polle A. Salinity tolerance of *Populus*. Plant Biology. 2010; 12(2): 317–333. doi: 10.1111/j.1438-8677.2009.00301.x 20398238 10.1111/j.1438-8677.2009.00301.x

[4] Wu B, Hu Y, Huo PJ, Zhang Q, Chen X, Zhang ZW. Transcriptome analysis of hexaploid hulless oat in response to salinity stress. PLoS ONE. 2017; 12(2): e0171451 doi: 10.1371/journal.pone.0171451 28192458 10.1371/journal.pone.0171451 PMC5305263

[5] Xu HG. Halophyte and ecological management of salinization in China Beijing: China Agricultural Science and Technology Press; 2004.

[6] Li JG, Pu LJ, Han MF, Zhu M, Zhang RS, Xiang YZ. Soil salinization research in China: advances and prospects. J Geogr Sci. 2014; 24(5): 943–960.

[7] Munns R, Tester M. Mechanisms of salinity tolerance. Annu Rev Plant Biol. 2008; 59: 651–681. doi: 10.1146/annurev.arplant.59.032607.092911 18444910 10.1146/annurev.arplant.59.032607.092911

[8] Turkan I, Demiral T. Recent developments in understanding salinity tolerance. Environ Exp Bot. 2009; 67(1):2–9.

[9] Deinlein U, Stephan AB, Horie T, Luo W, Xu GH, Schroeder JI. Plant salt-tolerance mechanisms. Trends Plant Sci. 2014; 19(6):371–379. doi: 10.1016/j.tplants.2014.02.001 24630845 10.1016/j.tplants.2014.02.001 PMC4041829

[10] Chartzoulakis KS. Salinity and olive: growth, salt tolerance, photosynthesis and yield. Agr Water Manage. 2005; 78(1–2): 108–121.

[11] Liu ZX, Zhang HX, Yang XY, Wei HR. Effects of soil salinity on growth, ion relations, and compatible solute accumulation of two sumac species: *Rhus glabra* and *Rhus trilobata*. Commun Soil Sci Plan. 2013; 44(21): 3187–3204.

[12] Cristiano G, Camposeo S, Fracchiolla M, Vivaldi GA, Lucia BD, Cazzato E. Salinity differentially affects growth and ecophysiology of two mastic tree (*Pistacia lentiscus* L.) accessions. Forests. 2016; 7: 156.

[13] Flowers TJ. Improving crop salt tolerance. J Exp Bot. 2004; 55(396): 307–319. doi: 10.1093/jxb/erh003 14718494 10.1093/jxb/erh003

[14] Munns R, James RA, Lauchli A. Approaches to increasing the salt tolerance of wheat and other cereals. J Exp Bot. 2006; 57(5): 1025–1043. doi: 10.1093/jxb/erj100 16510517 10.1093/jxb/erj100

[15] Cuartero J, Bolarín MC, Asíns MJ, Moreno V. Increasing salt tolerance in the tomato. J Exp Bot. 2006; 57(5):1045–1058. doi: 10.1093/jxb/erj102 16520333 10.1093/jxb/erj102

[16] Flowers TJ, Colmer TD. Salinity tolerance in halophytes. New Phytol. 2008; 179(4):945–963. doi: 10.1111/j.1469-8137.2008.02531.x 18565144 10.1111/j.1469-8137.2008.02531.x

[17] Cramer GR. Response of abscisic acid mutants of *Arabidopsis* to salinity. Funct Plant Biol. 2002; 29:561–567. 10.1071/PP01132 32689501

[18] Parrondo RT, Gosselink JG, Hopkinson CS. Effects of salinity and drainage on the growth of three salt marsh grasses. Bot Gaz. 1978; 139(1): 102–107.

[19] Yeo AR, Flowers TJ. Salt tolerance in the halophyte *Suaeda maritime* (L.) Dum.: evaluation of the effect of salinity upon growth. J Exp Bot. 1980; 31: 1171–1183.

[20] Chaves MM, Flexas J, Pinheiro C. Photosynthesis under drought and salt stress: regulation mechanisms from whole plant to cell. Ann Bot. 2009; 103(4): 551–560. doi: 10.1093/aob/mcn125 18662937 10.1093/aob/mcn125 PMC2707345

[21] James JJ, Alder NN, Muhling KH, Lauchli AE, Shackel KA, Donovan LA, et al High apoplastic solute concentrations in leaves alter water relations of the halophytic shrub, *Sarcobatus vermiculatus*. J Exp Bot. 2006; 57(1): 139–147. doi: 10.1093/jxb/erj016 16317037 10.1093/jxb/erj016

[22] Britto DT, Kronzucker HJ. Futile cycling at the plasma membrane: a hallmark of low-affinity nutrient transport. Trends Plant Sci. 2006; 11(11): 529–534. doi: 10.1016/j.tplants.2006.09.011 17035071 10.1016/j.tplants.2006.09.011

[23] Qiu NW, Yang CC, Lu ZK, Li ZN, Yue XJ, Cheng XX, et al Na^+^ compartmentation and physiological characteristics of *Artemisia anethifolia* in adaptation to saline environment. Acta Ecol Sin. 2014; 34(21): 6147–6155.

[24] Lv SL, Jiang P, Chen XY, Fan PX, Wang XC, Li YX. Multiple compartmentalization of sodium conferred salt tolerance in *Salicornia europaea*. Plant Physiol Bioch.2012; 51: 47–52. 10.1016/j.plaphy.2011.10.015 22153239

[25] Song J, Shi GW, Gao B, Fan H, Wang BS. Waterlogging and salinity effects on two *Suaeda salsa* populations. Physiol Plantarum. 2011; 141(4): 343–351. 10.1111/j.1399-3054.2011.01445.x 21214881

[26] Zhao KF, Song J, Feng G, Zhao M. Species, types, distribution, and economic potential of halophytes in China. Plant Soil. 2011; 342: 495–509.

[27] Yang S, Liu ZX, Zhang HX, Yang XY, Liu T, Yao ZG. Comprehensive evaluation of salt tolerance and screening identification indexes for three tree species. Scientia Silvae Sinica. 2013; 49(1): 91–98.

[28] Liu ZX, Zhang HX, Yang S, Yang XY, Di WB. Effects of NaCl stress on growth and photosynthetic characteristics of *Elaeagnus angustifolia* seedlings. Scientia Silvae Sinica. 2014; 50(1): 32–40.

[29] Liu ZX, Zhang HX, Yang XY, Liu T, Chen JH. Effects of Na_2_SO_4_ stress on growth and photosynthetic physiology of *Elaeagnus angustifolia* seedlings. Forest Research. 2014; 27(2): 186–194.

[30] Gong Q, Qiman Y, Ailijiang M. Effect of NaCl stress on matter accumulated and water content in the three species of oleaster plants seedlings. Journal of Xinjiang Agricultural University.2008; 31(3): 46–50.

[31] Ailijiang M, Qiman Y, Gong Q. Effects of NaCl stress on reactive-oxygen-scavenging enzymes and contents of osmotic adjustment substances in leaves of three species of oleaster plants seedlings. Xinjiang Agricultural Sciences.2008; 45(6): 1069–1075.

[32] Liu BY, Zhang WH, Liu XC, Jiang S. Salt tolerance of *Elaeagnus angustifolia* L. and *Caragana korshinskii* Kom. during germination. Bulletin Botanical Research. 2007; 27(6): 721–728.

[33] Tattini M, Remorini D, Pinelli P, Agati G, Saracini E, Traversi ML, et al Morpho-anatomical, physiological and biochemical adjustments in response to root zone salinity stress and high solar radiation in two Mediterranean evergreen shrubs, *Myrtus communis* and *Pistacia lentiscus*. New Phytol. 2006; 170(4): 779–794. doi: 10.1111/j.1469-8137.2006.01723.x 16684238 10.1111/j.1469-8137.2006.01723.x

[34] Bradford MM. A rapid and sensitive method for the quantitation of microgram quantities of protein utilizing the principle of protein-dye binding. Anal Biochem. 1976; 72: 248–254. 942051 10.1016/0003-2697(76)90527-3

[35] Zhang HX, Liu ZX, Liu QF. Seedling growth and salt tolerance of tree species under NaCl stress. Acta Ecol Sin, 2009; 29(5): 2263–2271.

[36] Abideen Z, Koyro HW, Huchzermeyer B, Ahmed MZ, Gul B, Khan MA. Moderate salinity stimulates growth and photosynthesis of *Phragmites karka* by water relations and tissue specific ion regulation. Environ Exp Bot.2014; 105: 70–76.

[37] Naidoo G, Naidoo Y, Achar P. Ecophysiological responses of the salt marsh grass *Spartina maritima* to salinity. Afr J Aquat Sci. 2012; 37(1):81–88.

[38] M'rah S, Ouerghi Z, Berthomieu C, Havaux M, Jungas C, Hajji M, et al Effects of NaCl on the growth, ion accumulation and photosynthetic parameters of *Thellungiella halophila*. J Plant Physiol. 2006; 163: 1022–1031. doi: 10.1016/j.jplph.2005.07.015 16971214 10.1016/j.jplph.2005.07.015

[39] Volkov V, Wang B, Dominy P, Fricke W, Amtmann A. *Thellungiella halophila*, a salt-tolerant relative of *Arabidopsis thaliana*, possesses effective mechanisms to discriminate between potassium and sodium. Plant Cell Environ.2004; 27: 1–14.

[40] Zhang Y, Sheng Y B, Luo X F. Effects of water stress on biomass and photosynthetic characteristics of Tetraploid Black Locust (*Robinia pseudoacacia* L.) clones. Forest Research. 2010; 23(4): 920–923.

[41] Grotkopp E, Rejmánek M, Rost TL. Toward a causal explanation of plant invasiveness: seedling growth and life-history strategies of 29 pine (*Pinus*) species. Am Nat. 2002;159: 396–419. doi: 10.1086/338995 18707424 10.1086/338995

[42] Wang SF, Hu YX, Li ZL, Sun HJ, Chen YT. Effects of NaCl stress on growth and mineral ion uptake, transportation and distribution of *Quercus virginiana*. Acta Ecol Sin. 2010; 30(17): 4609–4616.

[43] Ahmed MZ, Shimazaki T, Gulzar S, Kikuchi A, Gul B, Khan MA, et al The influence of genes regulating transmembrane transport of Na^+^ on the salt resistance of *Aeluropus lagopoides*. Funct Plant Biol. 2013; 40(8–9): 860–871. 10.1071/FP12346 32481156

[44] Jaarsma R, de Vries RSM, de Boer AH. Effect of salt stress on growth, Na^+^ accumulation and proline metabolism in Potato (*Solanum tuberosum*) cultivars. PLoS ONE. 2013; 8(3): e60183 doi: 10.1371/journal.pone.0060183 23533673 10.1371/journal.pone.0060183 PMC3606169

[45] Teakle NL, Tyerman SD. Mechanisms of Cl^-^ transport contributing to salt tolerance. Plant Cell Environ. 2010; 33(4):566–589. doi: 10.1111/j.1365-3040.2009.02060.x 19895402 10.1111/j.1365-3040.2009.02060.x

[46] Henderson SW, Baumann U, Blackmore DH, Walker AR, Walker RR, Gilliham M. Shoot chloride exclusion and salt tolerance in grapevine is associated with differential ion transporter expression in roots. BMC Plant Biol. 2014; 14: 273 doi: 10.1186/s12870-014-0273-8 25344057 10.1186/s12870-014-0273-8 PMC4220414

[47] Zhang WW, Yang HQ, You SZ, Fan SL, Ran K. MhNCED3, a gene encoding 9-*cis*-epoxycarotenoid dioxygenase in *Malus hupehensis* Rehd., enhances plant tolerance to Cl^-^ stress by reducing Cl^-^ accumulation. Plant Physiol Bioch. 2015; 89: 85–91. 10.1016/j.plaphy.2015.02.012 25725410

[48] Wei QJ, Gu QQ, Wang NN, Yang CQ, Peng SA. Molecular cloning and characterization of the chloride channel gene family in trifoliate orange. Biol Plantarum. 2015; 59(4): 645–653.

[49] Hasegawa PM, Bressan RA, Zhu JK, Bohnert HJ. Plant cellular and molecular responses to high salinity. Annu Rev Plant Physiol Mol Biol. 2000; 51(51): 463–499. 10.1146/annurev.arplant.51.1.463 15012199

[50] Gadallah MAA. Effects of proline and glycinebetaine on *Vicia faba* responses to salt stress. Biol Plantarum. 1999; 42(2): 249–257.

[51] Mullet JE, Whitsitt MS. Plant cellular responses to water deficit. Plant Growth Regul. 1996; 20(2): 119–124.

